# Optimization of Ambulatory Blood Pressure Monitoring during Pregnancy: A Path Toward Risk Stratification Improvement and Management of Hypertensive Disorders

**DOI:** 10.31083/RCM27235

**Published:** 2025-05-19

**Authors:** Yiwen Fang, Lushu Zuo, Jingge Li, Huihua Shi, Ruimin Zhang, Cha Han, Lijuan Lv, Xin Zhou

**Affiliations:** ^1^Department of Cardiology, Tianjin Medical University General Hospital, 300052 Tianjin, China; ^2^Department of Gynecology and Obstetrics, Tianjin Medical University General Hospital, 300052 Tianjin, China; ^3^Tianjin Key Laboratory of Female Reproductive Health and Eugenics, Tianjin Medical University General Hospital, 300052 Tianjin, China; ^4^Medical Genetic Center and Department of Obstetrics, Guangdong Women and Children Hospital, 511442 Guangzhou, Guangdong, China

**Keywords:** ambulatory blood pressure, pregnancy, diagnosis, prognosis

## Abstract

Hypertensive disorders of pregnancy (HDP) pose substantial risks to both maternal and fetal health, thereby highlighting the need for precise and comprehensive blood pressure (BP) monitoring methods. Ambulatory blood pressure monitoring (ABPM) offers advantages over traditional office BP measurements by enabling continuous 24-hour assessment, thus capturing circadian BP variations, including nocturnal and morning hypertension, which are often missed when BP is measured in a medical office. This capacity for detailed monitoring allows ABPM to identify specific BP phenotypes, such as normotension, white-coat hypertension, masked hypertension, and sustained hypertension. Each of these phenotypes has unique implications for risk stratification, which helps to identify high-risk pregnancies early and potentially improve outcomes through more targeted interventions. Despite these advantages, three key challenges have limited the widespread adoption of ABPM during pregnancy. First, the complex dynamics in BP variations throughout gestation are influenced by physiological adaptations, such as uterine artery remodeling, which lowers BP before 20 weeks and increases mean arterial pressure after 20 weeks to support fetal growth. Second, adaptive changes in the maternal arterial system alter vascular mechanical properties, complicating accurate BP assessments. Third, diagnostic thresholds specific to pregnancy that are directly linked to adverse pregnancy outcomes are lacking. Therefore, this review addresses the role of ABPM in managing HDP, examining BP dynamics and the suitability of monitoring devices, and ongoing efforts to develop diagnostic thresholds tailored to pregnancy. By exploring these aspects, this review underscores the importance of ABPM in advancing more precise, effective strategies for HDP management and multidisciplinary management programs for pregnant women to enhance clinical decision-making and maternal–fetal outcomes.

## 1. Introduction

As a leading cause of maternal morbidity and mortality worldwide, hypertensive disorders of pregnancy (HDP) affect 5–10% of pregnant women and their neonates [1, 2], which not only increases the risk of long-term maternal cardiovascular disease but also influences the occurrence of adverse events in offspring [3]. As HDP are defined by blood pressure (BP), the appropriate monitoring and management of BP are key in these disorders. Office blood pressure (OBP) measurement is the easiest available procedure in clinical practice and is limited in its presentation of BP variability [4]. Ambulatory blood pressure monitoring (ABPM) is a continuous 24-hour BP measurement technique that does not interfere with patients’ daily activities. BP values are recorded at specific intervals, which enables the calculation of BP indices such as the maximum, minimum, mean, and coefficient of variation, providing a comprehensive profile of BP patterns [5]. Therefore, ABPM offers a more comprehensive approach to BP monitoring than does OBP and demonstrates superior predictive and prognostic value for hypertension, cardiovascular disease, and mortality in nonpregnant populations [6, 7]. Other than OBP, four BP phenotypes can be identified, namely, normotension, white-coat hypertension (WCH), masked hypertension (MH) and sustained hypertension, which allows for better risk stratification. Another key advantage of ABPM is its ability to monitor BP continuously over a 24-hour period, which enables the detection of circadian patterns, including nocturnal and morning hypertension. Previous reviews [8, 9] have extensively described the diagnostic accuracy, thresholds, prognostic values and BP phenotypes based on ABPM in nonpregnant populations, which underscores its valuable role as a complement to OBP measurement.

Given the proven effectiveness of ABPM in guiding BP management in nonpregnant populations, along with the increasing focus on personalized medical care, recent guidelines have emphasized its role during pregnancy [10, 11]. However, three primary challenges limit the broader use of ABPM during pregnancy: (1) the complex dynamics of BP variations, (2) the adaptive changes in the mechanical properties of the maternal arterial system, and (3) the absence of pregnancy-specific diagnostic thresholds directly linked to adverse pregnancy outcomes (APOs). The first challenge complicates the timing of measurements, as BP fluctuations can vary significantly across different stages of pregnancy. The second challenge necessitates the use of BP devices that are specifically adapted to the physiological changes unique to pregnant women. The third challenge lies in the reliance on diagnostic thresholds derived from nonpregnant populations, as these thresholds focus on long-term cardiovascular risks rather than on APOs. This discrepancy increases the difficulty in the accurate interpretation of ABPM readings in the context of pregnancy, which highlights the need for thresholds that better reflect the relationship between BP levels and pregnancy-specific risks. This review addresses the implications of ABPM during pregnancy and examines dynamic BP changes, the suitability of detection devices, the ongoing efforts in developing diagnostic thresholds, and the relevance of BP phenotypes and derived parameters to prognosis. By highlighting these aspects, this review aims to support the development of more precise and effective approaches for the management of HDP that may lead to better clinical decision-making and improved pregnancy outcomes.

## 2. Physiological Characteristics of Blood Pressure during Pregnancy

**Fig. 1.**
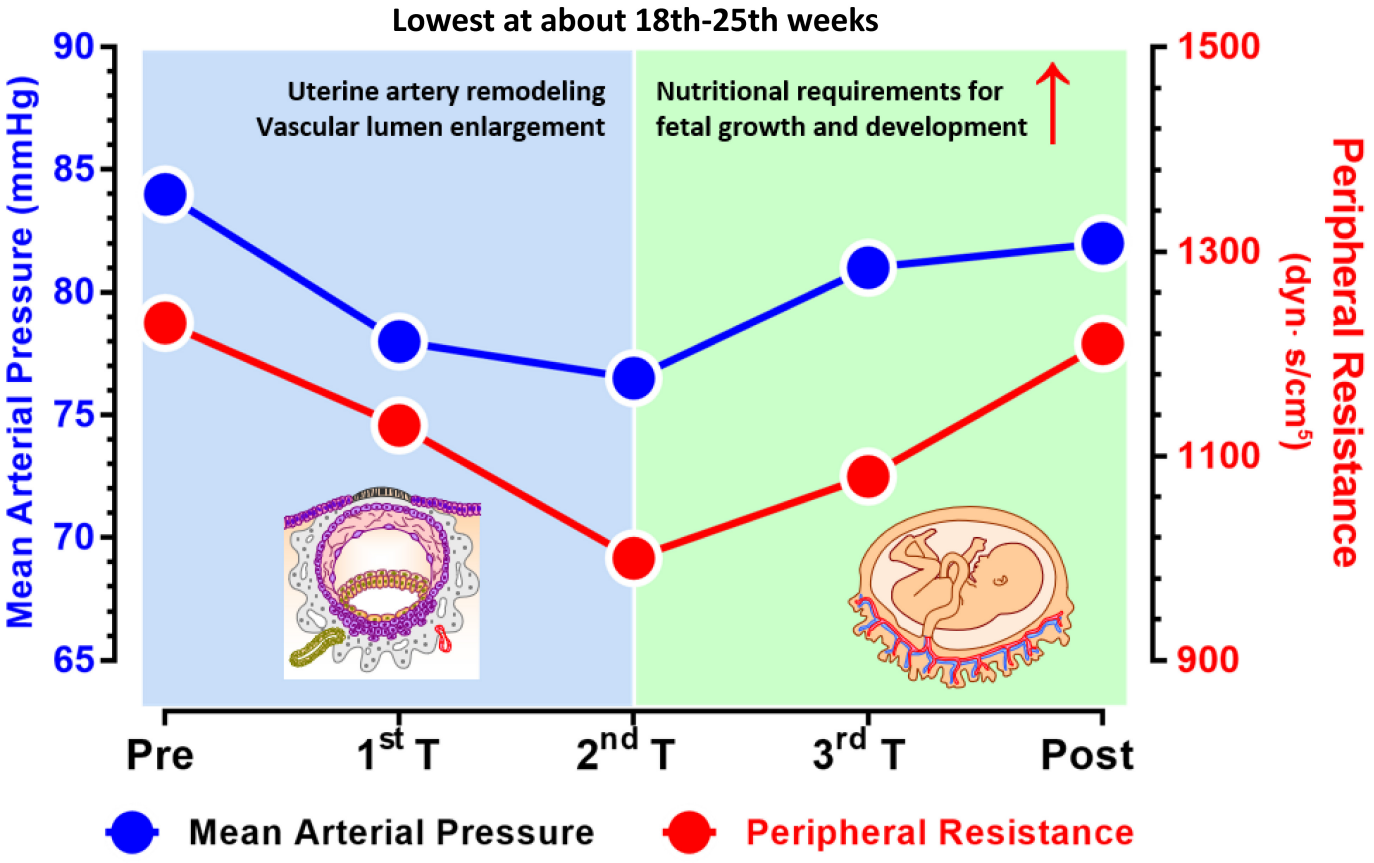
**Dynamic changes in the mean arterial pressure (left y-axis) and peripheral resistance during pregnancy (right y-axis)**. The mean arterial pressure varies in parallel with the peripheral resistance and are both the lowest in the middle of the second trimester (~20 weeks of gestation). Before approximately 20 weeks of gestation, physiological changes, such as uterine artery remodeling and vascular lumen enlargement, play a key role in reducing blood pressure. After 20 weeks, the rapid growth of the fetus and increased nutritional demands require an increase in the mean arterial pressure to support fetal development (figure is adapted with modifications from Curr Hypertens Rep. 2015; 17(5)) [17]. The figure was drawn using Prism 10 software (GraphPad Software, San Diego, CA, USA).

Pregnant women experience physiological and anatomical changes to adapt to the needs of increased metabolism and fetal growth during pregnancy. Regarding the cardiovascular system, there may be an increase in plasma volume, cardiac output, and arterial compliance, along with a reduction in peripheral resistance [12]. Specifically, in normal pregnancies, maternal blood volume increases by an average of 30–50% over the typical volume in nonpregnant individuals. This increase becomes noticeable at 6–8 weeks of gestation and continues to rise, peaking at approximately 32 weeks. In early pregnancy (1–12 weeks of gestation), the heart rate and stroke volume increase simultaneously. The former continues to gradually rise until term, whereas the latter plateaus at approximately 20 weeks of gestation. Moreover, systemic vascular resistance decreases, is lowest at approximately 20 weeks of gestation, and then gradually increases until term [13]. According to the dynamic nature of the cardiovascular system described above, BP levels tend to be “U-shaped” in normal pregnant women, as levels first decrease but then increase during pregnancy. Before mid-pregnancy (~20 weeks of gestation), the combined effects of increased blood volume and cardiac output and decreased systemic vascular resistance cause the BP to gradually decrease and reach its lowest level at approximately 20 weeks of gestation. Although the average time of the lowest point of systolic BP and diastolic BP varies depending on the baseline characteristics of each pregnant woman, these low points usually occur at approximately 18–22 weeks of gestation [14, 15]. After mid-pregnancy (~20 weeks of gestation) and until the end of gestation, to meet the needs of rapid fetal growth, it is not sufficient to rely solely on vascular regulation to maintain placental perfusion pressure, and maternal BP levels begin to increase. This moderate increase in BP may be a mechanism by which the placental blood supply and perfusion pressure are increased [16] (Fig. 1, Ref. [17]).

Women with multiple pregnancies tend to experience greater increases in cardiac output and heart rate [12]. Although BP changes follow a similar trend, women with multiple pregnancies tend to have higher BP levels than those with singleton pregnancies throughout the first, second, and third trimesters [18]. In addition, for women with HDP, different BP trajectories are observed depending on the specific subtypes of the disorder: women who develop gestational hypertension have higher BP levels, which may decrease more moderately before 20 weeks of gestation, and their BP levels may begin to rise earlier in mid-pregnancy, with a faster increase after 18 weeks of gestation [19, 20]. In comparison, the BP trajectory of patients with preeclampsia parallels that of normal pregnant women, but their BP levels are predominantly higher, with a steep increase in late mid-pregnancy, especially in cases of early-onset preeclampsia [19, 21]. The BP of women with chronic hypertension is higher than that of women with gestational hypertension and preeclampsia before 20 weeks of gestation but increases at a less rapid and dramatic rate after 30 weeks of gestation [20]. In other words, BP trajectories, in turn, reflect the type of HDP. Notably, BP trajectories may also be influenced by maternal factors such as body mass index, gestational weight gain and maternal habitual snoring [15, 22].

## 3. Ambulatory Blood Pressure Monitoring for Diagnosis

Considering the health and environmental impacts of mercury use in clinical settings, mercury-containing sphygmomanometers have been progressively phased out of clinical use [23]. In current medical practice, international guidelines [10, 11] recommend the use of certified and regularly calibrated upper-arm medical electronic sphygmomanometers for BP measurement. Moreover, compared with a single OBP measurement, ABPM provides a more accurate assessment of an individual’s BP in their daily life and significantly enhances the precision of BP measurements, which enables the identification of WCH and MH [24]. ABPM has also demonstrated superior predictive value, particularly for all-cause mortality and cardiovascular death [7]. Therefore, ABPM is prioritized for BP monitoring and management in clinical practice [11, 24].

ABPM is performed using automatic equipment, with the appropriate cuff size selected according to the individual’s arm circumference, according to the manufacturer’s instructions. Before wearing the cuff, initialization and installation of the equipment are necessary. After programming, BP levels can be recorded for at least 24 hours at preselected time intervals, and BP levels can usually be measured once every 20 minutes during the day and every 30 minutes at night. The daytime and nighttime periods are determined according to patients’ self-reported sleep and awake times. The requirement for valid monitoring is to have at least 20 valid daytime BP records or 7 nighttime BP records. If the above conditions are not met, the monitoring should be repeated [11, 24].

Importantly, the electronic BP monitors used routinely in nonpregnant populations rely on electronic technology and embedded algorithms for BP determination. However, these algorithms do not account for the alterations in hemodynamic and systemic arterial mechanical properties that occur during pregnancy, which may affect the accuracy of indirect BP measurements [25]. In patients with preeclampsia, BP measurements are usually underestimated due to specific pathological changes, including low arterial vascular compliance and increased interstitial edema [23]. Therefore, the use of BP monitoring in pregnant women (including those with preeclampsia) should undergo independent validation. A systematic review determined the accuracy of BP measurement devices in populations of pregnant individuals. Among the 28 devices examined, two ambulatory devices (BP lab and Welch Allyn QuietTrak) passed validation without any protocol violations [26]. More information regarding the type of device, applicable populations, and certification status can be obtained at https://www.stridebp.org and http://www.dableducational.org.

## 4. The Diagnostic Threshold of Ambulatory Blood Pressure for Hypertension

**Fig. 2.**
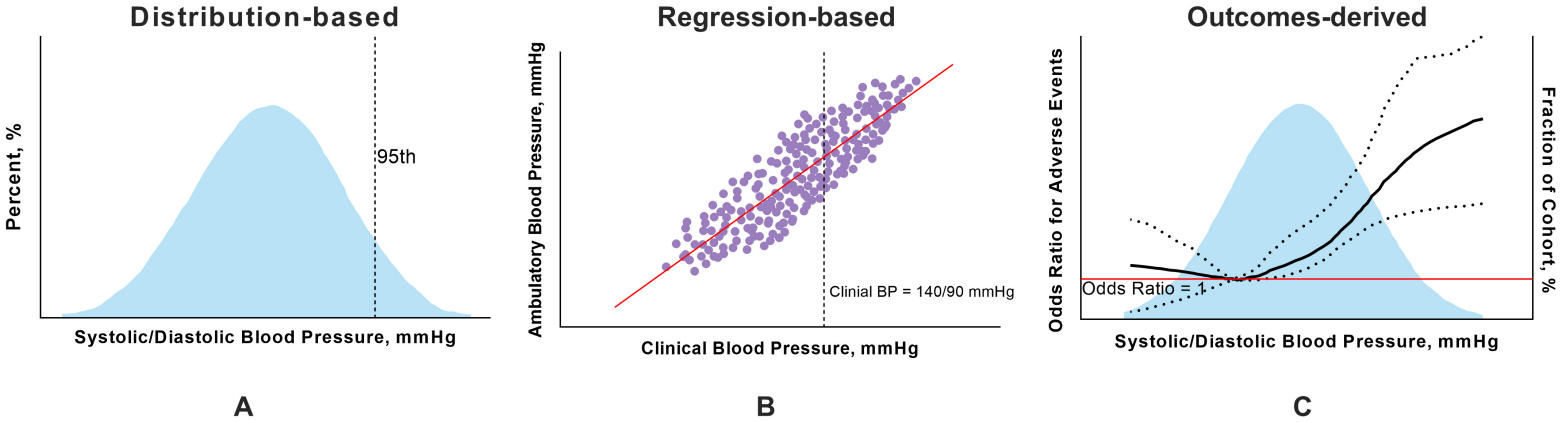
**Three main methods used to determine thresholds for ambulatory blood pressure monitoring**. (A) In the distribution-based approach, the values in the 95th percentiles are the thresholds for ABPM. (B) In the regression-based approach, the values of ambulatory BP corresponding to 140/90 mmHg for clinical BP are considered the diagnostic threshold for hypertension in ABPM. (C) In the outcome-derived approach, the restricted cubic spline shows a nonlinear correlation between ambulatory BP and APOs, and the threshold for ambulatory BP is the point with the lowest odds ratio. The figure was drawn using Prism 10 software (GraphPad Software, San Diego, CA, USA). ABPM, ambulatory blood pressure monitoring; APOs, adverse pregnancy outcomes; BP, blood pressure.

In the nonpregnant population, the current thresholds for the diagnosis of hypertension via ABPM are primarily based on average daytime, nighttime, and 24-hour average BP levels [9, 27, 28]. To determine BP thresholds for ABPM, a previous study has used three main methods: distribution-based, regression-based, and outcome-derived approaches [27]. The distribution-based method is used to obtain the percentiles (such as the 90th, 95th, and 99th percentiles) of the distribution of BP measurements obtained by ABPM, whereas the regression-based method correlates ABPM values with OBP values [29]. Notably, the outcome-derived method determines ABPM thresholds that align with OBP cutoff values, which provides equivalent predictions for future cardiovascular disease risk [30]. Although the thresholds set by different methods can vary, outcome-derived thresholds are generally considered more appropriate. As a result, the widely used diagnostic thresholds are based on this method. For example, clinical hypertension based on OBP measurements is defined as a BP ≥140/90 mmHg, and the corresponding ABPM thresholds, rounded to the nearest 0 or 5 mmHg, are set at 135/85 mmHg for the daytime, 130/80 mmHg for the 24-hour average, and 120/70 mmHg for the nighttime. These thresholds have been shown to predict a similar 10-year cardiovascular risk as the OBP threshold of 140/90 mmHg [30] (Fig. 2).

Ravenell and colleagues employed the aforementioned methods to establish slightly different ABPM thresholds for black adults [28], which provided cardiovascular disease or all-cause mortality risks similar to risks predicted by clinical BP thresholds; this reflects the unique cardiovascular risk profile of this population. Nevertheless, the pregnant population presents more specific characteristics than the control population. Therefore, establishing pregnancy-specific diagnostic thresholds for ABPM is essential. Previous studies have applied nonoutcome-derived methods to identify normal upper limits of gestational-specific ABPM thresholds during pregnancy [31, 32]. However, this approach does not align with the methodology of utilizing outcome-derived thresholds for nonpregnant adults. Considering that the primary risk factors associated with hypertension during pregnancy focus on APOs rather than on long-term cardiovascular risks, it is inappropriate to apply hypertension diagnostic criteria derived from nonpregnant populations to pregnant women. Our previous work determined the “optimal” ABPM thresholds for pregnant women at high risk for HDP and for those who were diagnosed with HDP in late pregnancy. After rounding to the nearest 0 or 5 mmHg, the outcome-derived, clinically unrelated thresholds identified were 130/80 mmHg for the daytime, 120/75 mmHg for the nighttime, and 130/75 mmHg for the 24-hour average, whereas the outcome-derived, clinically-relevant BP thresholds were 135/85, 125/80, and 135/85 mmHg for the daytime, nighttime and 24-hour averages, respectively. When a nonoutcome-derived approach was applied, the thresholds for daytime, nighttime and 24-hour averages were 135/85, 130/80 and 135/85 mmHg, respectively [33]. Although the pregnancy-specific thresholds we investigated are derived from APOs and determined using a thorough methodology, these thresholds were constrained because ABPM was conducted exclusively during the third trimester. Given the dynamic changes in BP during pregnancy, it is essential to establish specific diagnostic thresholds for both ABPM and OBP that are tailored to each stage of pregnancy; this includes the determination of the optimal timing for ABPM. This would allow for more accurate identification of high-risk pregnant women and enable the implementation of individualized BP management strategies in a cost-effective manner.

## 5. Blood Pressure Phenotypes Based on Ambulatory Blood Pressure Monitoring

### 5.1 White-Coat Hypertension

WCH refers to elevated OBP that occurs before 20 weeks of gestation but is normal in settings outside of a medical office. In the nonpregnant population, the prevalence of WCH reported in previous studies varies from 20% to 30% [34, 35]. Due to differences in BP measurement protocols and diagnostic criteria, the prevalence of WCH during pregnancy also varies and ranges from 4% to 30% [36, 37]. Notably, the association between WCH and APOs is controversial; for example, a meta-analysis revealed that WCH may increase the risk of preeclampsia, preterm birth, and delivery of small for gestational age (SGA) neonates in pregnant women, which indicates a greater risk of developing HDP [36, 38]. However, another study revealed no statistically significant difference in the development of preeclampsia or neonatal mortality between pregnant individuals with WCH and those with normotension, which indicates that the prognosis of WCH during pregnancy may be relatively benign [39]. Additionally, a persistent status of WCH until delivery is associated with a lower risk of SGA than preeclampsia or gestational hypertension. Moreover, pre-pregnancy WCH is more closely related to higher birthweight and a lower rate of thrombocytopenia than sustained hypertension [40]. It is undeniable that patients with WCH present a greater likelihood of developing gestational hypertension, the association of which with APOs has been well documented. ABPM is recommended for those with OBPs ≥140/90 mmHg before 20 weeks of gestation to diagnose and manage white coat hypertension [10]. Once a diagnosis of WCH is confirmed, extensively repeated BP measurements are warranted to assess the aforementioned risks, identify the need for antihypertensive medication and avoid overdiagnosis and overtreatment [41].

### 5.2 Masked Hypertension

MH refers to BP that is normal when obtained in a medical office before 20 weeks of pregnancy but that is elevated when measured in settings outside of a medical office. The prevalence of MH in the pregnant population has not been well defined. Previous studies have shown that approximately 30% of high-risk pregnant women have MH and that the prevalence is approximately 20% among untreated normotensive pregnant women [42, 43, 44]. Women with MH have a 6.8-fold greater risk of developing preeclampsia than women with normal BP [43]. Moreover, MH is an independent predictor of the development of preeclampsia and adverse neonatal outcomes [42, 43]. Therefore, further screening of preeclampsia risk during antenatal care can be performed in combination with clinical BP measurements and ultrasonographic and laboratory parameters [45]. Compared with WCH, MH is more challenging to detect because the OBP readings are normal [41]. Recognizing this difficulty, a study by Wu *et al*. [46], which involved a low-risk cohort of 47,874 participants, demonstrated that when systolic/diastolic BP levels are between 130–139/80–89 mmHg in early pregnancy, it is crucial to perform out-of-office BP measurements to rule out MH. Similarly, the study by Salazar *et al*. [47] showed an increased risk of developing preeclampsia in high-risk pregnant women with OBPs ≥125/75 mmHg. Therefore, the use of ABPM in appropriate populations may help identify individuals who appear to be at high risk but whose risks may not be fully captured through standard OBP measurements.

### 5.3 Nocturnal Hypertension

Nocturnal hypertension is also a relatively common phenomenon and is defined as ABPM ≥120/70 mmHg during nighttime rest [24, 48]. This definition was also used in previous studies on gestational nocturnal hypertension [49, 50]. In essence, nocturnal hypertension is a type of masked condition [43, 51]. A meta-analysis of 17,312 patients with hypertension confirmed the significance of nocturnal hypertension in the prognosis of sustained hypertension and MH [52]. The mechanism by which nocturnal hypertension contributes to MH may be secondary to increased sympathetic nervous system activity [53]. Generally, individuals with factors such as sleep deprivation, obstructive sleep apnea, and metabolic syndrome predominantly exhibit nocturnal hypertension [51]. Therefore, when major and moderate risk factors for preeclampsia (PE) are considered, abnormal sleep behavior should not be ignored. Nocturnal hypertension and MH are frequently observed in pregnant women. Our previous work [54] explored the risk of hypertension phenotypes for APOs and APO-related office and ambulatory BP thresholds. In a cohort of 967 high-risk pregnant women, the prevalences of nocturnal hypertension and MH were 25.5% and 11.4%, respectively. Among women with MH, 95.5% presented with nocturnal hypertension [54], which indicates a strong correlation between them and suggests that even patients with a normal 24-hour average BP obtained by ABPM may present with isolated nocturnal hypertension. Moreover, women with MH and nocturnal hypertension are likely to experience placental ischemia and abnormal uterine spiral artery remodeling [55]. Placental ischemia can trigger a cascade of short-term and long-term maternal pathological events. An imbalance of soluble fms-like tyrosine kinase-1 and placental growth factor (PIGF) in women with preeclampsia can lead to vascular damage and nocturnal hypertension, which may further exacerbate placental ischemia and impair remodeling of the placental spiral arteries [56, 57]. Additionally, Chen and colleagues reported a negative correlation between serum PIGF levels and nocturnal BP, which indicates a specific association between nocturnal BP and PIGF [58]. Notably, even after delivery, nocturnal hypertension and vascular damage may persist and lead to an increased short- and long-term risk of cardiovascular disease [59]. Hence, the use of ABPM for the detection of nocturnal hypertension and to control BP during pregnancy is imperative for improved maternal and fetal health.

### 5.4 Morning Hypertension

A previous study revealed that morning hypertension is common even in patients with well-controlled OBP [60]. Morning hypertension has been linked to increased cardiovascular risk [61] and may increase the risk for stroke in the elderly [62]. However, research on morning hypertension during pregnancy is limited. As mentioned earlier, the diagnostic threshold of ABPM in the general population includes BP levels based on daytime, nighttime and 24-hour averages. Hence, we advocate for additional research focused on the exploration of the associations among morning hypertension, which may be related to isolated daytime hypertension, uncontrolled nocturnal hypertension and adverse pregnancy outcomes. This research is not intended to complicate hypertension management in pregnant women, but rather, to emphasize the importance of identifying abnormal BP phenotypes to enable more tailored and effective management strategies, such as optimization of the timing of antihypertensive medication.

### 5.5 Transient Gestational Hypertension

In contrast to that regarding WCH, research on transient gestational hypertension, which is specifically characterized by its onset in early pregnancy, is limited. Several studies have defined transient hypertension as BP that is elevated in the second and third trimesters of pregnancy (usually after 20 weeks of gestation) that then returns to normal in subsequent BP assessments, and some measurements even recover within several hours [10, 63]. However, another study noted a similar phenomenon in which BP was temporarily elevated in early pregnancy but returned to normal at 14–19 weeks [64]. Although the increase in BP is only temporary, this increase is associated with an increased risk of developing true gestational hypertension and preeclampsia before delivery despite the stage at which the increase occurs [63, 64].

## 6. Blood Pressure Circadian Rhythm

The BP circadian rhythm was calculated as the (daytime BP – nighttime BP)/daytime BP × 100% and was categorized into five patterns: extremely dipping (nighttime BP decreases by >20% of the daytime level), dipping (decrease by 10–20%), non-dipping (decrease by 0–10%), and reverse dipping (nighttime BP decrease <0%). This BP circadian rhythm is influenced by both intrinsic and extrinsic factors. In young hypertensive individuals, the non-dipping BP pattern was found to be associated with an increased risk of cardiovascular disease, including nonfatal myocardial infarction, stroke and heart failure. Combined with BP variability, the non-dipping pattern provides better prognostic performance than does the average 24-hour ambulatory BP level [65]. The BP circadian rhythm of nonpregnant and pregnant patients is similar but is different in patients with chronic hypertension and preeclampsia [66]. Individuals with HDP show an average blunted dipping pattern in the second trimester compared with normotensive individuals according to consecutive nighttime BP monitoring [67], which may serve as a potential predictor of HDP severity. In preeclampsia, the acrophase of BP shifts to nighttime hours, whereas the peak typically occurs in the afternoon in normotensive individuals [68], which contributes to the non-dipping or even reverse-dipping pattern observed in severe preeclampsia [66]. Moreover, the non-dipping BP pattern was found to be associated with maternal hemodynamic changes, including reduced longitudinal velocity, decreased cardiac output and lower diastolic velocity of the mitral valve annulus [69]. The potential mechanism behind these abnormal BP variability patterns may be related to conditions such as diabetes mellitus, which affects the autonomic nervous system, or sleep apnea, which leads to intermittent hypoxia. The severity of maternal obstructive sleep apnea-hypopnea has been reported to be inversely correlated with a greater decline in nocturnal BP [70]. Pregnant women with gestational diabetes mellitus and obesity have a higher incidence and earlier onset of non-dipping BP patterns during pregnancy [71]. Interestingly, pregnant women with a history of childhood adversity display alterations in the hypothalamic-pituitary-adrenal axis and immune activity, contributing to the loss of the typical nocturnal BP decline [72]. A study has reported on the circadian rhythm of BP during pregnancy, most of which have focused on two main patterns, dipping and non-dipping patterns. Unfortunately, these patterns may persist into the postpartum period, as nearly half of HDP women exhibit a non-dipping pattern [73], along with diastolic dysfunction. Severe and recurrent preeclampsia have been identified as significant predictors for non-dipping pattern in the postpartum period [74].

## 7. Prognostic Values of Ambulatory Blood Pressure Monitoring for Long-Term Maternal and Fetal Outcomes

The clinical value of ABPM during pregnancy has been thoroughly discussed above. As a pregnancy complication, preeclampsia characterized by target organ damage [75] and fetal growth restriction, significantly increases both the short-term and long-term risk of cardiovascular disease and all-cause mortality [76], which necessitates medical supervision and individualized interventions during the postpartum period. More than 80% of individuals with HDP experience ongoing hypertension after delivery, and approximately 14% develop severe hypertension [77]. The most common postpartum hypertension phenotype is MH, followed by sustained hypertension and WCH [73]. A previous study [78] compared the BP profiles between women with preeclampsia and those with normotension at 6–12 weeks postpartum and revealed that 17.9% of pregnant women experienced complications associated with MH, which is linked to sustained hypertension and an increased risk of cardiovascular disease [79]. In another study, 64.5% of patients experienced complications associated with nocturnal hypertension, which increased the risk of cardiovascular disease and stroke [80]. These findings highlight the critical need for continuous blood pressure monitoring postpartum. A similar pattern extends to 1 year after severe preeclampsia [73], and MH continues at a similar rate, whereas nocturnal hypertension affects 42.5% of women.

The predictive value of ABPM for infant outcomes has focused primarily on birthweight during pregnancy, with a stronger association with SGA than with office BP measurements [81], which is potentially independent of maternal BP elevation [82]. At 28 weeks of gestation, the average diastolic BP was negatively correlated with both head circumference and birthweight [83], as well as with admission to the neonatal intensive care unit after delivery [84], which demonstrates the strongest predictive performance of all ambulatory BP parameters [85]. Moreover, the offspring of mothers with early-onset preeclampsia exhibit higher nocturnal systolic BP values than those with late-onset preeclampsia 6 weeks after delivery, and this abnormal BP profile persists into childhood and typically continues until the ages of 6–12 years [86].

## 8. Knowledge Gap and Future Directions 

**Fig. 3.**
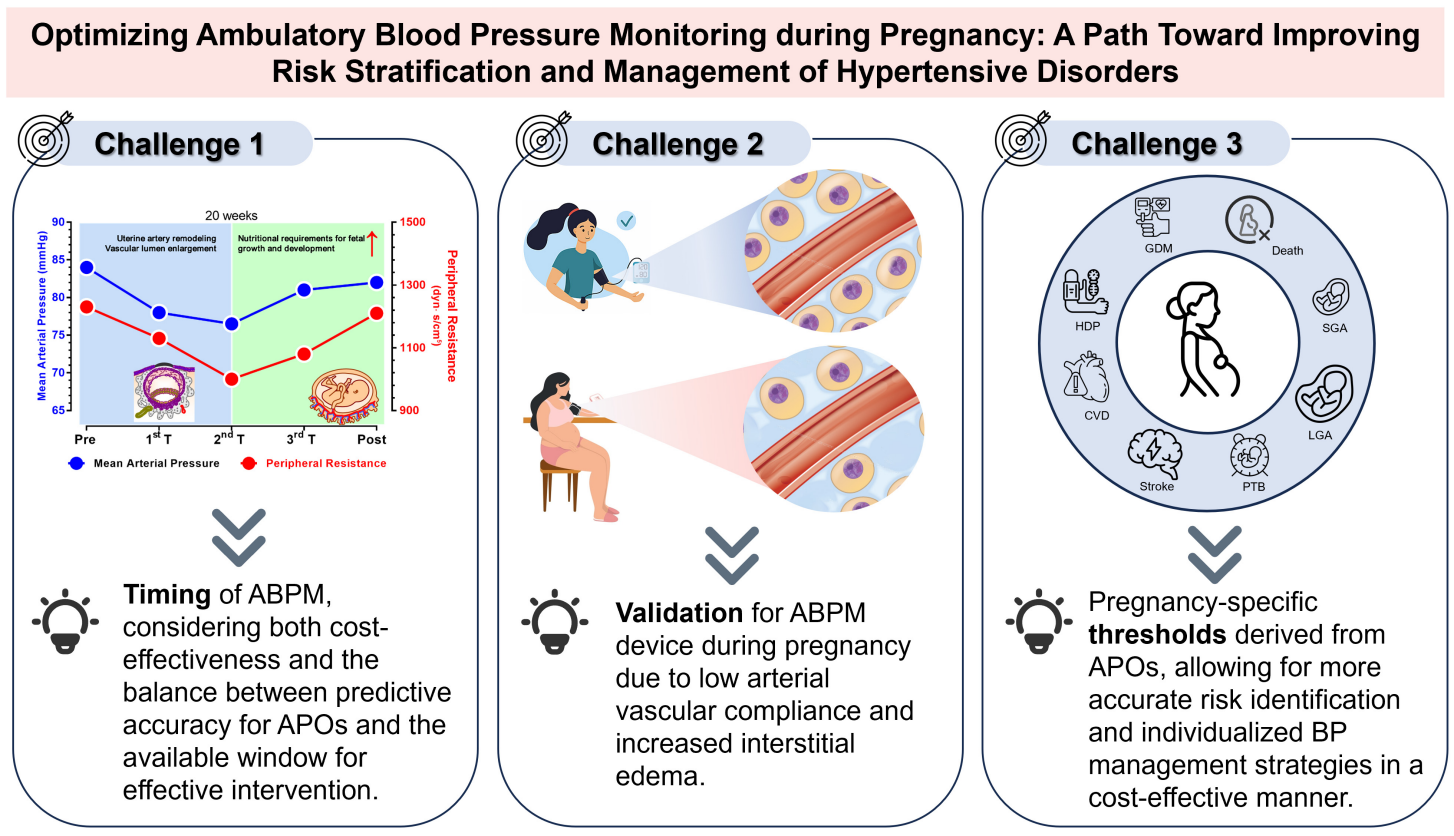
**Three main challenges of future ABPM research**. The figure was drawn using Prism 10 software (GraphPad Software, San Diego, CA, USA).

Due to three primary challenges, namely, the complex dynamics of BP variations, the adaptive changes in the mechanical properties of the maternal arterial system, and the absence of pregnancy-specific diagnostic thresholds directly linked to APOs, ABPM has not been widely adopted in gynecological practice. The following knowledge gaps and research directions should be addressed and considered. First, multidisciplinary management of pregnant women is warranted. ABPM holds significant potential—perhaps with the aid of machine learning models—not only to improve predictive capabilities for HDP but also to recommend the most appropriate treatment. However, its use must be prescribed and interpreted by professionals with expertise not only in BP but also in pregnancy and obstetric pathology. This underscores the importance of multidisciplinary care and the critical role of obstetric medicine, as emphasized in recent papers and guidelines on the subject [87, 88, 89, 90]. Second, the optimal timing for ABPM during pregnancy remains unclear. While current guidelines recommend out-of-office BP monitoring before 20 weeks for an accurate diagnosis of WCH and chronic hypertension, the optimal timing for ABPM after 20 weeks has not yet been explored. This timing should consider both cost-effectiveness and the balance between predictive accuracy for APOs and the window available for effective intervention. Third, clinicians and others should advocate for the certification of BP measurement devices specifically for use in pregnant women, particularly those with HDP. Fourth, large longitudinal studies across multiple gestational stages with ethnically diverse populations are needed to establish pregnancy outcome-derived ABPM diagnostic thresholds, as well as outcome-derived thresholds for in-office BP measurements. Such studies should focus on standardized diagnostic thresholds that appropriately balance the risks of adverse maternal and neonatal outcomes. Additionally, future research should explore the relationship between ABPM and composite APOs, including perinatal death, intracranial hemorrhage, and respiratory distress in neonates. Moreover, with technological advances, several novel cuffless wearable devices and smartphone applications have emerged [91], which offer professional healthcare solutions and increase the ease by which individuals can stay connected with medical providers. These innovations may simplify ABPM and provide a broader platform for its use (Fig. 3).

## 9. Conclusion

ABPM has the potential to enhance risk stratification in patients with HDP by providing a more detailed understanding of BP dynamics. Future efforts to develop pregnancy-specific diagnostic thresholds and determine the optimal timing for ABPM are crucial steps that can enable timely interventions and improve outcomes for both mothers and their offspring. By refining these approaches, ABPM can become a key tool in the effective management of high-risk pregnancies.

## Abbreviations

ABPM, ambulatory blood pressure monitoring; APOs, adverse pregnancy outcomes; BP, blood pressure; HBPM, home blood pressure monitoring; HDP, hypertensive disorder of pregnancy; MH, masked hypertension; OBP, office blood pressure; PIGF, placental growth factor; SGA, small for gestational age; WCH, white coat hypertension.
